# Shared deficits in space, time, and quantity processing in childhood genetic disorders

**DOI:** 10.3389/fpsyg.2013.00043

**Published:** 2013-02-07

**Authors:** Carmelo M. Vicario, Mark J. Yates, Michael E. R. Nicholls

**Affiliations:** ^1^School of Psychology, University of QueenslandBrisbane, QLD, Australia; ^2^Department of Psychology, University of MelbourneParkville, VIC, Australia; ^3^School of Psychology, Flinders UniversityAdelaide, SA, Australia

In the last decade, there has been a gradual consolidation of the idea that the processing of time, space and quantity arise from common neural mechanisms. This model, formally described as “A Theory Of Magnitude” (ATOM) (Walsh, 2003), suggests that the mechanism underlying the overlap in the neuro-cognitive processing of magnitudes is mediated by the activity of a special pool of neurons localized in the frontal and parietal cortices. These neurons may work by making analogical inferences among magnitudes, probably by extracting their covariant factors (Vicario and Martino, 2010).

While lesion (Critchley, 1953; Oliveri et al., 2009), neuroimaging (Dehaene et al., 1998; Rao et al., 2001) and cognitive research (Fischer et al., 2003; Vicario et al., 2007, 2008, 2009, 2011; Loetscher et al., 2008; Loftus et al., 2008; Oliveri et al., 2009; Renzi et al., 2011; Vicario, 2011, 2012; see Bonato et al., 2012 for a complete review) can be used to support the ATOM model, genetic conditions, such as Turner syndrome, Fragile X syndrome, and Chromosome 22q11.2 deletion syndrome have received relatively little attention. These conditions are particularly interesting because they all appear to affect mathematical cognition. For example, kindergarten girls with Turner syndrome have difficulty relative to their peers on aspects of mathematics, such as the automaticity of fact retrieval and timed calculations, and make more procedural errors during problem-solving (Rovet, 1993; Temple and Marriott, 1998). Children with Fragile X syndrome have more difficulties than their peers on aspects of applied counting, such as the ability to enumerate (e.g., one-to-one correspondence) and the identification of the *n*th item in a set (Murphy et al., 2006). Finally, individuals with Chromosome 22q11.2 deletion syndrome are significantly slower at counting the number of elements in a set and perform more poorly than control participants on a number comparison task (De Smedt et al., 2006, 2009).

In addition to mathematical deficits, these genetic conditions also affect a broad range of cognitive abilities relating to the perception and processing of space and time. In relation to Turner syndrome, females perform more poorly than controls on tests of spatial ability. Deficits were also reported for the serial processing ability, which requires intact temporal processing ability (Silbert et al., 1977). These spatial and temporal deficits, which have been replicated by subsequent researchers (Mazzocco et al., 2006; Murphy and Mazzocco, 2008; Beaton et al., 2010), also seem to extend to visuoattentional functioning (Hart et al., 2006).

Abnormalities in spatio-temporal visual processing have also been reported in childhood Fragile X syndrome. For instance, infants with Fragile X syndrome show significantly reduced sensitivity to the detection of texture-defined dynamic stimuli, although they are capable of detecting static patterns and luminance-defined dynamic patterns at the same level as age-matched controls (Farzin et al., 2008). Studies using behavioral psychophysics in adult males with Fragile X syndrome corroborate this view by reporting a reduced contrast sensitivity for visual stimuli presented at high temporal frequencies (Kogan et al., 2004). Moreover, Farzin et al. (2011) have demonstrated a drastically reduced resolution of temporal visual attention in this syndrome, which was directly linked to the extent of the genetic trinucleotide repeat mutation in the FMR1 gene.

In similar fashion, children with Chromosome 22q11.2 deletion syndrome show cognitive impairments in spatiotemporal processing and visuospatial attention mechanisms (Simon, 2008). In the context of this syndrome, it is interesting that Simon (2008) noted that the impairment of mathematical abilities in these children arises from a decreased representational resolution documented for both space and time (Simon et al., 2005a,b), which he named “spatiotemporal hypergranularity” (Simon, 2008, p. 2). Spatiotemporal hypergranularity implies that the mental representations of spatiotemporal cognitive functions in children with 22q11.2DS have a coarser resolution, possibly as a consequence of lower resolution of spatial attention functions in this population (Simon, 2008).

Data from brain imaging might help to disclose the “*fil rouge*” binding these three pathologies. The link in question could be alterations in the frontal and parietal regions of these patients.

Neuroanatomical studies on Turner patients have consistently found decreased gray matter volume in the bilateral parietal lobes, parieto-occipital region, and subcortical gray matter (Murphy et al., 1993; Reiss et al., 1995; Brown et al., 2002; Good et al., 2003; Kesler et al., 2003; Molko et al., 2003). A study by Holzapfel et al. (2006) has also documented lower fractional anisotropy values in the deep white matter of the left parietal-occipital region extending anteriorly along the superior longitudinal fasciculus into the deep white matter of the frontal lobe.

Evidence for disordered activity in the fronto-parietal network in Fragile X syndrome has also been reported. Yang et al. (2012) showed decreased parietal P3 amplitude and diminished fronto-central positivities with a delayed onset (50 ms later than controls) during an auditory oddball task requiring dual responses. Moreover, two studies in females with Fragile X have examined brain function during working memory (Menon et al., 2000) and mental arithmetic (Rivera et al., 2002) tasks. These studies showed that, in parallel with performance deficits, females with Fragile X also had altered activity in frontoparietal networks (in both hemispheres) associated with these tasks.

Finally, different studies on children affected by Chromosome 22q11.2 deletion syndrome have reported reductions in gray and white matter corresponding to parietal (Eliez et al., 2000; Kates et al., 2001; Simon et al., 2005a,b; Campbell et al., 2006) and frontal lobes (Kates et al., 2004), as well as an altered connectivity involving the fronto-parietal network in this childhood disorder (Simon, 2008). Moreover, Srivastava et al. (2012) reported atypical cortical gyrification, which was mainly distributed along the medial aspect of each hemisphere in 6–15-year-old children with 22q11.2DS. Significantly, these cortical areas included parietal structures that are associated, in typical individuals, with visuospatial and attentional functioning, and numerical and temporal cognition.

While Turner Syndrome, Fragile X Syndrome, and Chromosome 22q11.2 deletion syndrome are all childhood genetic disorders, the different syndromes do have very different genetic etiologies, developmental trajectories, and physical manifestations. It is remarkable, therefore, that the syndromes appear to affect an overlapping set of cognitive processes. Mathematical ability is most probably one of the best known outcomes of the genetic syndromes. From the evidence reviewed in this paper, however, it also clear that the children suffer from a similar set of processing problems related to time and space. Although the link between mathematics and spatial/temporal processing is a subject of debate, recent work with children with developmental dyscalculia show that these children also have problems with temporal (Sigmundsson et al., 2010; Vicario et al., 2012) and spatial attentional (Ashkenazi and Henik, 2010) processing. There is therefore tantalizing evidence suggesting a causal link between mathematical ability and spatial/temporal processing, which deserves further investigation.

The association between the functions most probably arises from damage to a common set of neural mechanisms. Turner Syndrome, Fragile X Syndrome, and Chromosome 22q11.2 deletion syndrome all appear to affect circuits located in the parietal and frontal cortices. Neural circuits lying in these same regions are also thought to play an important role in the ATOM model (Walsh, 2003) and provide a common code for numbers, space, and time. Variations in the extent and exact nature of the deficits may be related to the specific structures affected by each of the genetic disorders.

The current paper has provided a brief overview of childhood genetic disorders which affect the frontal and parietal cortices. The children with these various disorders also appear to suffer from an overlapping set of cognitive disturbances. While the overlap between Turner syndrome, Fragile X syndrome, and Chromosome 22q11.2 deletion syndrome is compelling, more work needs to be done. A comparative test, applying the same tests of temporal and spatial processing ability, across the different genetic conditions would allow more powerful conclusions to be drawn.
